# Should perioperative immunonutrition for elective surgery be the current standard of care?

**DOI:** 10.1093/gastro/gow008

**Published:** 2016-04-14

**Authors:** Shishira Bharadwaj, Brandon Trivax, Parul Tandon, Bilal Alkam, Ibrahim Hanouneh, Ezra Steiger

**Affiliations:** ^1^Center for Human Nutrition, the Cleveland Clinic Foundation, Cleveland, OH, USA; ^2^College of Osteopathic Medicine, Michigan State University, East Lansing, MI, USA

**Keywords:** immunonutrition, infections, postoperative complications, cost-effectiveness, guidelines

## Abstract

Postoperative infectious complications are independently associated with increased hospital length of stay (LOS) and cost and contribute to significant inpatient morbidity. Many strategies such as avoidance of long periods of preoperative fasting, re-establishment of oral feeding as early as possible after surgery, metabolic control and early mobilization have been used to either prevent or reduce the incidence of postoperative infections. Despite these efforts, it remains a big challenge to our current healthcare system to mitigate the cost of postoperative morbidity. Furthermore, preoperative nutritional status has also been implicated as an independent risk factor for postoperative morbidity. Perioperative nutritional support using enteral and parenteral routes has been shown to decrease postoperative morbidity, especially in high-risk patients. Recently, the role of immunonutrition (IMN) in postoperative infectious complications has been studied extensively. These substrates have been found to positively modulate postsurgical immunosuppression and inflammatory responses. They have also been shown to be cost-effective by decreasing both tpostoperative infectious complications and hospital LOS. In this review, we discuss the postoperative positive outcomes associated with the use of perioperative IMN, their cost-effectiveness, current guidelines and future clinical implications.

## Introduction

Postoperative infectious complications are a major contributor to increased inpatient morbidity, hospital length of stay (LOS) and cost [1]. Approximately 54% of all hospital-acquired infections occur in the postoperative phase [2]. Furthermore, postoperative surgical infections have resulted in US$1.6 billion in excess healthcare costs per year and extended hospital LOS by roughly 1 million days per year according to the 2005 hospital stay data from the Nationwide Inpatient Sample [3]. Also, a recent study of 1442 patients, which investigated the risk factors for hospital readmissions within 30 days of an index hospitalization, reported the most common reasons to be gastrointestinal (GI) problem/complication (28%), surgical infection (22%) and failure to thrive/malnutrition (10%) [4]. Surgical procedures associated with higher rates of readmissions included pancreatectomy, colectomy and liver resection. Additionally, blood transfusion, postoperative pulmonary complication, wound complication, sepsis/shock, urinary tract infection and vascular complications were associated with increased risk of readmission [4].

Historically, there has been a positive correlation between malnutrition and increased risk of infections [5]. Additionally, protein calorie malnutrition occurs in up to 90% of surgical oncology patients [6]. Malnutrition along with surgical stress predisposes patients to significant postoperative complications and immune depression [7,8]. Lymph node atrophy, decreased total lymphocyte population and dysfunctional cellular immunity associated with malnutrition increase postoperative infection rates [7,8]. These processes are greatly implicated in patients with GI malignancies undergoing surgical resection [9,10]. Therefore, strategies have been implemented to circumvent malnutrition such as avoiding preoperative fasting, re-establishing oral feeding as soon as possible and maintaining good glycemic and metabolic control. Furthermore, research is now demonstrating the importance of additional perioperative nutritional support through the use of enteral and parenteral feeds in high-risk patients [11].

In the past, several studies reported deficiencies in amino acids such as arginine and glutamine in the postoperative period [12,13]. Arginine was then supplemented in supraphysiological concentrations in the postoperative phase, resulting in significant reduction in postoperative complications and infection rates [12,13]. This led to the concept of implementing immunomodulating diets or immunonutrition (IMN) to counteract postoperative immune depression and improve overall clinical outcome in surgical patients [14–16]. Through improved cellular immunity, neutrophil phagocytic activity and increased total lymphocyte counts, IMN has been shown to reduce postoperative infectious complications, decrease hospital LOS and improve nutritional status in critically ill patients [15–17]. In the past, IMN was believed to be beneficial only in malnourished patients. However, recent studies have shown that this belief is a misconception, and in fact both nourished and malnourished patients may benefit from IMN [15–18].

In this review, we aim to discuss the physiology of specific constituents of immune-enhancing diets. We also present a comprehensive analysis of several randomized controlled trials (RCTs), systematic reviews and meta-analyses comparing preoperative, perioperative and postoperative IMN in surgical patients. Finally, we discuss the cost-effectiveness, current guidelines and future clinical implications of IMN.

### Major components of the immunonutrition formula

IMN is defined as enteral nutritional formulas supplemented with some combination of arginine, glutamine, omega-3 fatty acids and nucleotides with the goal of improving host immune response and ing. These substrates have been found to positively modulate postsurgical immunosuppression and inflammatory response.

#### Arginine

Arginine is a conditionally essential amino acid that is synthesized in the body but not in concentrations high enough to meet the requirements for metabolic needs and support during periods of stress such as surgery, trauma or growth [19]. Under normal physiologic conditions, the immune system uses very little arginine [20]. However, while under stress, arginine is the primary fuel source for T cells; therefore, it helps maintain immune function and decreases the risk of infection [20–22]. Zhu *et al.* demonstrated that T-cell proliferation was linearly dependent on plasma arginine concentration with CD8+ T cells being slightly more sensitive to arginine deprivation than CD4+ T cells [21,22]. Hence, L-arginine may become deficient in times of extreme stress such as the postoperative period.

The mechanism by which arginine modulates immune function has been well studied. Nitric oxide synthase (NOS) utilizes arginine as a source for nitric oxide (NO) production [23,24]. NO deficiency has been well-correlated with wound breakdown and poor wound healing [13,20]. Furthermore, NO increases oxygenation and microperfusion by causing vasodilation [23,24]. Vasodilation with subsequent increase in oxygenation has a positive effect on the body’s ability to fight infection through the recruitment of leukocytes and macrophages. Also, macrophages use L-arginine as a substrate for many of their immune functions. Additionally, NO has inherent bactericidal activity [24]. However, NO’s effect on vasodilation is concerning in septic shock patients, and therefore arginine is cautiously used in this specific subset of patients [13,20].

L-arginine can also be metabolized by arginase-1 into ornithine and urea in order to produce hydroxyproline. Hydroxyproline has been implicated in wound healing and connective-tissue growth [25,26]. Amplication of arginase-1 reaction increases in the presence of escalations in T helper cells-2 (Th2) cytokine production and after major elective surgery [13,20]. Additionally, arginase-1 activity has been found to be up-regulated by myeloid-derived suppressor cells, which are immature cells made in the bone marrow that develop after major surgery or post trauma [27,28]. These cells have been shown to be major producers of arginase-1 enzyme and therefore suppress the immune system by diminishing arginine availability to T cells and NOS after major surgery or post trauma requiring supplementation [27,28]. Arginine also promotes collagen synthesis and growth hormone production, which suggests its role in wound healing [29].

#### Omega-3 fatty acids

Omega-3 fatty acids are long-chain polyunsaturated fatty acids that have a well-established effect on the immune system. Eicosapentaenoic acid (EPA) and docosahexaenoic acid (DHA) are two major types of omega-3 fatty acids that have been shown to lower levels of arachidonic acid (AA) and increase the production of resolvins and protectins, which play a role in the resolution of inflammation and enhance wound healing [30,31]. Furthermore, EPA and DHA enhance immune response by improving lymphocyte function [32]. While the interaction between arginine and omega-3 fatty acids is incompletely understood, omega-3 fatty acids may decrease the expression of arginase-1 [33]. Bansal *et al.*, using prostaglandin-E3 (PG-E3) from fish oil (a well-known source of omega-3 fatty acids) and interleukin-13 (IL-13, a Th2 cytokine inducer) reported that arginase-1 induction was significantly lower in cells that received PG-E3 in the presence of IL-13 [33].

#### Nucleotides

Nucleotides are low molecular-weight intracellular compounds, which are the building blocks of ribonucleic acid (RNA) and deoxyribonucleic acid, play a key role in nearly all biochemical processes. They are essential for rapidly replicating cells such as T cells for their maturation, proliferation and function [34,35]. Also, dietary nucleotide supplementation has been shown to increase villus heights, mucosal proteins and brush border enzymes in the GI tract [36]. While the role that nucleotides play with arginine and other components of the IMN mechanism remains largely unclear, Yamauchi *et al.* demonstrated that arginine does stimulate *in vitro* nucleotide synthesis [37].

#### Glutamine

Glutamine is a conditionally essential amino acid that plays a crucial role in B-cell differentiation, neutrophil superoxide production, cytokine production, T-cell proliferation and phagocytosis [38,39]. Glutamine is a major source of energy for proliferation and differentiation of enterocytes [38]. Similar to arginine, glutamine concentrations are markedly decreased in inflammatory states and hence may have a suppressive effect on the immune system [40].

## Current evidence

Multiple meta-analyses and RCTs have investigated the role of IMN in the preoperative, perioperative and postoperative phases in surgical oncology patients. The evidence supporting the use of IMN in this patient population has been controversial with the majority of studies, however, reporting a reduction in postoperative complications and LOS.

Several components of IMN including arginine, omega-3 fatty acids and nucleotides directly modulate the immune status of surgical patients. Suzuki *et al.* prospectively studied the quantified T-cell response in 30 patients who underwent pancreaticoduodenectomy and received either perioperative oral IMN or postoperative oral IMN or postoperative parenteral nutrition (PN) [41]. Postoperatively, patients who received perioperative IMN had increased levels of messenger RNA expression levels of T-bet, interferon-gamma, related orphan receptor gamma and IL-17F compared with other groups. Furthermore, the rate of infectious complications was significantly reduced in the perioperative group compared with other groups. IMN has also been shown to cause an increase in B-lymphocyte fraction [42], increase in the CD4/CD8 ratio [43], decrease in postoperative lymphocyte count [44], higher serum EPA and EPA/AA ratio [45] and higher preoperative monocytic expression of HLA-DR epitopes [46].

### Preoperative immunonutrition

In the preoperative phase, formulas enriched with arginine, omega-3 fatty acids and nucleotides have been shown to improve postoperative immune response, gut oxygenation and intestinal microperfusion [15,16,47–50]. Furthermore, preoperative IMN reduces the overall infection rate, hospital LOS and postoperative complications. In well-nourished colorectal surgical patients, Braga *et al.* demonstrated that patients who received preoperative IMN had a postoperative infection rate of 12% compared with 32% in patients who received isoenergetic/isonitrogenous formula [15]. Additionally, the average hospital LOS was 12 days in the control group compared with 9.5 days in the IMN group. Furthermore, by use of polarographic probes and laser Doppler flowmetry, the investigators reported improvement in gut oxygenation and intestinal microperfusion in those patients. Additionally, in another prospective, randomized, double-blind study, Braga *et al.* demonstrated a decline in postoperative infection rate from a baseline of 42% in patients who received standard isocaloric/ isonitrogenous tube feeds to 28% in patients who received IMN preoperatively [16]. The hospital LOS decreased by 2.1 days in the preoperative IMN group compared with the control group. Similarly, Gianotti *et al.* also studied the effect of IMN in well-nourished gastroesophageal, pancreatic and colorectal cancer surgical patients who received either preoperative IMN, perioperative IMN or no nutritional support [49]. The authors reported a decrease in the incidence of postoperative infections and hospital LOS in both the preoperative and perioperative IMN groups compared with the control. Similar results were also reported in cardiac surgical patients [46].

Other studies, however, have found conflicting results on the use of preoperative IMN [51–55]. McCarter *et al.* concluded that preoperative supplementation with arginine-only diets or arginine + omega-3 fatty acid diets for 7 consecutive days showed no improvement in lymphocyte mitogenesis or clinical outcomes [51]. Similarly, in another study of 42 patients with GI tumors supplemented with arginine, omega-3 and RNA, there was no decrease in postoperative complications, hospital LOS and overall outcomes compared with the control group [52]. A larger prospective trial of 244 patients who underwent elective total gastrectomy also concluded that a 5-day preoperative enteral IMN diet did not have any effect on postoperative complication rates [55]. However, the aforementioned studies included well-nourished patients. Hence, the effect of IMN therapy may be more beneficial in patients who are malnourished, which was reported by Barker *et al.*, who demonstrated a non-significant decrease in hospital LOS in malnourished GI surgery patients [50]. Therefore, further studies may be needed to delineate the exact role of IMN and whether differences exist in regards to patients’ underlying nutritional status.

### Perioperative immunonutrition

Similar to the preoperative phase, perioperative supplementation with arginine, omega-3 fatty acids and nucleotides may also result in fewer postoperative complications, shorter hospital LOS and improved overall clinical outcome [56–58]. In a prospective, randomized study of 154 patients who underwent elective upper GI surgery, perioperative enteral IMN resulted in a significant decrease in postoperative infectious complications (14 *vs* 27; *P* = 0.05) as well as overall postoperative complications (7 *vs* 16; *P* = 0.04) compared with the control group who received an isoenergetic diet [8]. Similarly, Braga *et al.* prospectively studied the effect of 7 days of IMN or isoenergetic control diets in 206 patients who underwent elective gastric, colorectal or pancreatic surgery [10]. Furthermore, jejunal infusions were commenced postoperatively. A 16% decrease in postoperative infection rates and a significant decline in overall hospital LOS were observed. Klek *et al.* investigated 305 gastric and pancreatic surgical patients who were randomized to receive either a 14-day perioperative IMN including arginine, omega-3 fatty acids and glutamine or a standard diet [58]. In addition to the reduction in infectious complications and decreased LOS, the study investigators found a significant decrease in mortality in the IMN group compared with the control group.

The role of perioperative IMN has also been studied in non-GI surgical patients. A study by Snyderman *et al.* investigated the role of IMN in head and neck cancer patients [56]. One hundred thirty-six patients were randomly assigned to receive one of four treatment groups: preoperative/postoperative IMN, postoperative IMN, preoperative/postoperative standard formula and postoperative standard formula. The authors reported a significant decrease in the incidence of postoperative infectious complications in patients who received IMN compared with control. However, no significant difference in wound-healing problems or duration of hospitalization was seen in both groups. Furthermore, Celik *et al.* examined the role of perioperative IMN in gynecological oncology patients [57]. After receiving 9 consecutive days of IMN (2 days preoperative and 7 days postoperative), white blood cell count, lymphocyte population and CRP levels increased significantly in the IMN group compared with control. Furthermore, the rates of wound infections, hospital LOS and overall complications were significantly lower in the IMN group.

There have been few studies that have, however, reported no benefit in perioperative IMN supplementation [59–62]. In malnourished head and neck cancer patients undergoing surgery, the role of perioperative arginine supplementation on immune status and postoperative outcome was examined [59]. In this prospective randomized study, 49 patients were randomly assigned to receive either no preoperative and standard postoperative tube feeding, standard preoperative and postoperative tube feeding or arginine-supplemented preoperative and postoperative tube feeding. The authors reported no significant improvement in nutritional status, surgery-induced immune suppression or clinical outcome. Similarly, Finco *et al.* studied the effect of perioperative enteral IMN in patients undergoing laparoscopic colorectal surgery [60]. Twenty-eight patients were randomized to either diet enriched with arginine, omega-3 fatty acids and RNA or a low-fiber diet. Although, there was an increase in CD4 lymphocytes on the day before surgery as compared with baseline parameters (*P* < 0.05) in the IMN group, there was no significant difference between the two groups in postoperative infectious complications. Interestingly, Klek *et al.* investigated the role of IMN without L-arginine supplementation [61]. Two hundred fourteen well-nourished patients who underwent upper GI surgery were randomly assigned to receive either standard enteral nutrition, immunomodulating enteral nutrition or standard parenteral nutrition. The authors reported no statistically significant reduction in infectious complications or hospital LOS. The lack of arginine supplementation in IMN diet in those patients could have played a role. Additionally, Tepaske *et al.* found that the addition of glycine to perioperative IMN formula in high-risk cardiac surgery patients did not add any additional benefit in overall postoperative inflammatory response [62].

### Postoperative immunonutrition

Immune-enhancing diets have also been shown to have significant benefit if started postoperatively [63–70]. Daly *et al.* investigated 60 adult patients who underwent surgery for upper GI cancer [63]. An increase in omega-3/omega-6 ratios and a decrease in PG-E2 production was observed with postoperative IMN. Additionally, mean hospital LOS decreased by 6 days, and overall postoperative infectious complications declined by 33% in response to the immune-enhancing diet. Similarly, another study of 85 patients who underwent surgical resection of upper GI cancer reported significantly fewer infectious and wound complications (11% *vs* 37%; *P* = 0.02) and shorter mean hospital LOS (15.8 +/- 5.1 days *vs*. 20.2 +/- 9.4 days) in the supplemented group than in the standard group [64]. In a separate study, Marano *et al.* randomized 109 gastrectomy patients to receive either postoperative IMN or an isocaloric-isonitrogenous control nutrition diet within 6 hours of surgery [70]. The incidence of postoperative infectious complications (7% *vs* 20%; *P* < 0.05) including anastomotic leak rate (4% *vs* 7%, *P* <0.05) was lower in the IMN group compared with control.

Similar results have also been reported in patients undergoing non-GI surgeries, particularly head and neck oncology patients [65–69]. Casas-Rodero *et al.* randomized 44 oral and laryngeal cancer patients undergoing head and neck surgery postoperatively to one of the three groups: an arginine-enhanced formula; a standard polymeric formula or an arginine, RNA and omega-3 fatty acids enhanced formula [69]. Postoperative infectious complications were significantly less frequent in the group that received immune-enhanced enteral nutrition formulas. However, length of postoperative stay and rates of fistula formation did not differ between the two groups. Similarly, Riso *et al.* postoperatively randomized 44 head and neck cancer patients to receive either IMN or standard isocaloric/isonitrogeneous control diet [65]. A significant reduction in postoperative infections and overall hospital LOS was observed in malnourished patients. Also, an increase in total lymphocyte population was observed on postoperative days 4 and 8 in the IMN group.

Few studies, however, have reported no benefit of IMN supplementation in the postoperative phase [71–73]. Braga *et al.* randomly allocated 78 patients who underwent major abdominal surgeries for gastric/pancreatic cancers to either standard enteral diet or diets enriched with arginine, omega-3 and RNA [71]. The authors concluded that there was no statistically significant difference in rates of postoperative infections between the 2 groups, although the severity of infection was milder in the IMN group compared with control. Similarly, in a prospective study, 130 upper GI cancer patients who underwent pancreatic, esophageal or gastric resection were randomly assigned to receive either an immune-enhancing diet or isonitrogeneous/isocaloric diet postoperatively [72]. There was no change in overall clinical outcome in the IMN group compared with the control.

### Meta-analyses

Numerous meta-analyses have investigated the effectiveness of IMN in reducing overall postoperative infectious complications and hospital LOS (Table 1) [74–82]. Zheng *et al.*, in their meta-analysis of 13 RCTs including 1269 GI cancer patients, compared perioperative IMN formula with standard oral diet [74]. The authors concluded that IMN decreased postoperative infection rates and hospital LOS, although it did not show survival benefit. They also found increased total lymphocyte counts, IgG levels, CD4+ counts and decreased IL6 levels in patients who received IMN patients compared with standard diet. Similarly, another meta-analysis of 35 RCTs (25 RCTs with GI surgeries and 10 RCTs with non-GI surgeries) including patients who underwent major elective surgery for GI, head and neck, cardiac and gynecological malignancy reported a decline in postoperative infection rates by 41%, and hospital LOS decreased by 2.38 days when patients were given arginine-supplemented IMN [75]. Additionally, IMN was found to be effective in both GI and non-GI surgical patients. Furthermore, combined arginine, omega 3- fatty acids and nucleotide formulas showed a significant benefit over arginine formulas alone. Also, perioperative administration of IMN showed the greatest benefit compared with preoperative or postoperative IMN. In contrast, another meta-analysis of 17 RCTs by Waitzberg *et al.*, including gastrointestinal, cardiac and head and neck surgical patients who were prescribed IMPACT (arginine, omega-3 fatty acids, and nucleotides) reported lower rates of postoperative infection and a decreased LOS of 3.1 days per patient regardless of the time of IMN administration [76]. While the reduction in infections and hospital LOS was established, no mortality benefit was reported with the use of IMN, consistent with previous results. In a separate meta-analyses of 21 studies including 1918 patients, the majority with GI malignancy, Marik *et al.* reported reduced risk of acquired infections, wound complications and reduced hospital LOS in high-risk patients [77]. Furthermore, the authors found that the benefits of IMN required the use of formulas that contained both arginine and fish oil than either alone. Furthermore, the timing of IMN did not influence their results. This systematic review provides evidence for the synergistic relationship between arginine and omega-3 fatty acids. The above studies demonstrate a significant benefit of IMN in reducing postoperative infectious complications and hospital LOS. However, a direct survival benefit has yet to be reported.

**Table 1.** Meta-analyses of studies on the role of immunonutrition in surgical oncology patients

Meta-analyses	Number of randomized control trials	Type of surgery	Commercial formula used	Outcomes	Effect on mortality
Zheng *et al.* [74]	13	Gastrointestinal surgeries	Not specified	Fewer postoperative infections, shorter hospital length of stay, increase in lymphocyte and CD4 counts, increase in IgG levels, decrease in IL-6 levels	No effect
Drover *et al.* [75]	35	Gastrointestinal, surgery, cardiac, head and neck, gynecological surgeries	IMPACT, NUTRISON INTENSIVE, STRESSON, RECONVAN	Fewer postoperative infections, shorter hospital length of stay (except with fewer GI surgeries)	No effect
Waitzberg *et al.* [76]	17	Gastrointestinal, cardiac, head and neck	IMPACT	Fewer postoperative infections, shorter hospital length of stay, fewer anastomotic leaks	No effect
Marik *et al.* [77]	21	Gastrointestinal, surgery, cardiac, head and neck	IMPACT, STRESSON	Fewer postoperative infections, shorter hospital length of stay	Not reported
Cerantola *et al.* [78]	21	Gastrointestinal surgeries	Not specified	Fewer overall complications, Fewer postoperative infections, shorter hospital length of stay (except when given preoperatively)	No effect
Marimuthu *et al.* [79]	26	Gastrointestinal surgeries	IMPACT, STRESSON, RECONVAN and ALITRA	Fewer infectious complications, fewer noninfectious complications (with postoperative administration), shorter hospital length of stay (with perioperative and postoperative administration)	No effect
Zhang *et al.* [80]	19	Gastrointestinal surgeries	Not specified	Fewer postoperative infections, shorter hospital length of stay, Fewer noninfectious complications (when IMN administered perioperatively)	Not reported
Osland *et al.* [81]	21	Gastrointestinal surgeries	IMPACT, NUTRISOURCE, STRESSON, RECONVAN, INTESTAMIN	Fewer postoperative infections, shorter hospital length of stay (peri+postoperative administration), Fewer noninfectious complications (postoperative administration), fewer anastomotic leaks (perioperative administration)	No effect
Hegazi *et al.* [82]	15	Gastrointestinal surgeries	Not specified	No effect on postoperative infections or hospital length of stay	Not reported

### *Is immunonutrition cost*-*effective?*

A great deal of attention is currently being focused on cost-effectiveness of certain interventions. Despite improved surgical techniques, postoperative morbidity and its related costs are a major burden to any healthcare system. There has been constant search for interventions to mitigate or offset the cost of postoperative morbidity. There is significant debate whether the cost of IMN products would be cost-effective by reducing the postoperative complications and length of stay. Several studies using economic models have tried to investigate this issue and have found this strategy to be cost-effective. One study, which investigated the postoperative morbidity cost in GI cancer patients using hospital billing system and Medicare costs, reported that a surgical infection added US$12 542 (length of hospital stay (37% of costs, 26% of charges), laboratory testing (22% of costs, 25% of charges), radiology tests (9% of costs, 4% of charges), pharmaceuticals (7% of costs, 10% of charges) and use of other hospital services (24% of costs, 35% of charges)) to the cost of patient care [83]. Patients with postoperative fever but without documented infection were also more expensive to care for than afebrile uninfected patients and added US$9145 to the cost of care. The authors reported that strategies should be implemented to define more efficient management strategies in the care of patients with postoperative fevers with and without documented infection without compromising the care of patients. Another prospective randomized multicenter study, which included 154 GI cancer patients, reported a lower postoperative complication rate (14 *vs* 27; *P* = 0.05) and cost-effectiveness of IMN compared with control (1503 Deutsche Marks (DM) *vs* 3587 DM) [8]. In a separate study, which included data from 126 member hospitals and more than 1 million surgical, medical and trauma patients reported an economic benefit of US$2066 in medical patients and US$688 and US$308 per patient in surgical and trauma patients with the use of IMN [84]. Another study of 206 cancer patients who received either perioperative IMN or standard enteral nutrition found a cost-effectiveness of €2386 per complication-free patient in patients treated with IMN compared with standard enteral diet [85]. Similarly, another prospective study, which randomized 305 well-nourished GI cancer patients to either 5-day preoperative IMN or conventional treatment, found an economic advantage for IMN (€6245 *vs* €2985) compared with the conventional group with respect to postoperative morbidity [86]. Furthermore, the mean cost of postoperative complications was €4492, of which anastomotic leaks, abdominal abscesses and pancreatic fistulae and wound infections were the most common. Another study, which investigated cost reduction based on the Waitzberg meta-analysis, found a cost benefit of US$3300 in infectious complications and US$6000 on hospital LOS in patients who were given perioperative IMN [87]. Despite the limitations of these studies including the difference in economic parameters depending on the healthcare system between different countries, reimbursement rates, type of surgery and complication rates, IMN is cost-effective by reducing both the incidence of postoperative infections and hospital LOS.

### Current guidelines

The American Society of Parenteral and Enteral Nutrition (ASPEN) recommends that patients who undergo major neck or abdominal cancer surgery, trauma, burns or are critically ill and on mechanical ventilation receive enteral formulations that are supplemented with arginine, glutamine, nucleic acid, omega-3 fatty acids and antioxidants [88]. However, ASPEN does caution the use of these enteral formulations in patients with severe sepsis. The European Society of Parenteral and Enteral Nutrition (ESPEN) recommends the use of IMN formulas in malnourished patients undergoing major neck and abdominal cancer surgery [89]. Additionally, ESPEN recommends that IMN should commence before surgery and continue for 5–7 days postoperatively. In 2012, the North American Surgical Nutrition Summit laid down consensus recommendations for the use of IMN in surgical patients [90]. These included a greater emphasis on preoperative metabolic preparation and optimizing health status, performing preoperative nutritional risk assessments, perioperative IMN, considering carbohydrate loading preoperatively and the use of protocols to implement the appropriate surgical nutritional interventions. Furthermore, the IMN protocol includes administering 500–1000 ml of IMN formula containing arginine, omega-3 fatty acids and nucleotides per day for 5 days preoperatively followed by at least 1000 kcal of IMN formula per day for 5 days postoperatively.

### Clinical implications and future research

Studies are in uniform agreement that IMN improves postoperative infection rates, lowers complications and shortens LOS in elective surgical patients. The reason for no survival benefit in these patients could be due to the low mortality rate in patients undergoing elective surgery. Furthermore, these benefits are more pronounced when IMN is given perioperatively *vs* preoperatively or postoperatively. However, there is heterogeneity in these studies with respect to the types of IMN formulas used, type of surgery, underlying nutritional status, timing of administration, control group and mode of IMN administration. Future studies should focus on standardizing the dosage, timing and components of IMN formulas, use for different patient populations including non-cancer surgical patients and investigating whether IMN formulas have greater benefit in malnourished patients compared with well-nourished patients.

## Summary

Major elective surgery involves extreme stress on the body that results in unique immunological and inflammatory responses regardless of the underlying nutritional status. IMN given perioperatively has been extensively studied to show benefit in lowering infection rates and overall complications and shortening hospital LOS. Furthermore, the cost-effectiveness of such an intervention can provide significant savings in the current rising healthcare costs. Hence, IMN may be considered current standard of care in patients undergoing elective surgery.

*Conflict of interest statement*: none declared.
